# Plant and Soil Enzyme Activities Regulate CO_2_ Efflux in Alpine Peatlands After 5 Years of Simulated Extreme Drought

**DOI:** 10.3389/fpls.2021.756956

**Published:** 2021-10-14

**Authors:** Zhongqing Yan, Enze Kang, Kerou Zhang, Yong Li, Yanbin Hao, Haidong Wu, Meng Li, Xiaodong Zhang, Jinzhi Wang, Liang Yan, Xiaoming Kang

**Affiliations:** ^1^Beijing Key Laboratory of Wetland Services and Restoration, Institute of Wetland Research, Chinese Academy of Forestry, Beijing, China; ^2^Sichuan Zoige Wetland Ecosystem Research Station, Tibetan Autonomous Prefecture of Aba, Zoige, China; ^3^University of Chinese Academy of Sciences, Beijing, China; ^4^Information Center of Ministry of Ecology and Environment, Beijing, China

**Keywords:** alpine peatland, extreme drought, ecosystem respiration, soil respiration, extracellular enzyme activities

## Abstract

Increasing attention has been given to the impact of extreme drought stress on ecosystem ecological processes. Ecosystem respiration (Re) and soil respiration (Rs) play a significant role in the regulation of the carbon (C) balance because they are two of the largest terrestrial C fluxes in the atmosphere. However, the responses of Re and Rs to extreme drought in alpine regions are still unclear, particularly with respect to the driver mechanism in plant and soil extracellular enzyme activities. In this study, we imposed three periods of extreme drought events based on field experiments on an alpine peatland: (1) early drought, in which the early stage of plant growth occurred from June 18 to July 20; (2) midterm drought, in which the peak growth period occurred from July 20 to August 23; and (3) late drought, in which the wilting period of plants occurred from August 23 to September 25. After 5 years of continuous extreme drought events, Re exhibited a consistent decreasing trend under the three periods of extreme drought, while Rs exhibited a non-significant decreasing trend in the early and midterm drought but increased significantly by 58.48% (*p* < 0.05) during the late drought compared with the ambient control. Plant coverage significantly increased by 79.3% (*p* < 0.05) in the early drought, and standing biomass significantly decreased by 18.33% (*p* < 0.05) in the midterm drought. Alkaline phosphatase, polyphenol oxidase, and peroxidase increased significantly by 76.46, 77.66, and 109.60% (*p* < 0.05), respectively, under late drought. Structural equation models demonstrated that soil water content (SWC), pH, plant coverage, plant standing biomass, soil β-D-cellobiosidase, and β-1,4-N-acetyl-glucosaminidase were crucial impact factors that eventually led to a decreasing trend in Re, and SWC, pH, β-1,4-glucosidase (BG), β-1,4-xylosidase (BX), polyphenol oxidase, soil organic carbon, microbial biomass carbon, and dissolved organic carbon were crucial impact factors that resulted in changes in Rs. Our results emphasize the key roles of plant and soil extracellular enzyme activities in regulating the different responses of Re and Rs under extreme drought events occurring at different plant growth stages.

## Introduction

There are growing pieces of evidence that extreme drought events will occur more frequently and at greater intensity under the predicted climate change caused by a mass release of carbon dioxide (CO_2_), (Dai, 2013; Asadieh and Krakauer, 2015). More extreme drought events affect the key processes of ecosystem functions, such as vegetation photosynthesis (Deng et al., 2021b), plant community composition (Zang et al., 2020), ecosystem water-use efficiency (Wang et al., 2021), and circulation of nutrients (Zhou et al., 2016; Schönbeck et al., 2020). Furthermore, drought has particularly severe effects on pools and fluxes (Hao et al., 2018) in terrestrial carbon (C) cycles (Schlesinger et al., 2016; Ren et al., 2017), which may cause positive C-climate feedback. A deeper understanding of the impact of these extreme drought events on C dynamics in natural ecosystems around the world is necessary (Deng et al., 2021a).

Ecosystem respiration and soil respiration (Rs) play a significant role in the maintenance of the functions and sustainability of terrestrial ecosystems (Chen et al., 2019; Zhou et al., 2019). Ecosystem respiration (Re) is the total sum of soil microbial, root, leaf, and stem respiration (Beer et al., 2010). Rs represents CO_2_ released into the atmosphere from the soil surface, including that from autotrophic respiration of living roots and symbionts (Zhou et al., 2014), as well as microbial respiration in soil organic matter and plant litter decomposition (Bond-Lamberty and Thomson, 2010). Relevant results of field observations and modeling show that the annual variation in atmospheric CO_2_ is strongly affected by the variation in Re and Rs caused by climate change (Kato et al., 2004). Therefore, small fluctuations in Re and Rs may induce remarkable variations in CO_2_ concentrations in the atmosphere (Luo, 2007; Metcalfe et al., 2011). While Re and Rs are known to be controlled under multiple biotic and abiotic factors, understanding how Re and Rs respond to extreme drought and the driving mechanism is of great significance for predicting C-climate feedback over regional to global scales.

Plant productivity and species composition are critical to peatland ecosystem C sequestration and soil C input, and high-water levels and associated saturated soils play a key role in maintaining peatland vegetation composition and productivity (Bakker et al., 2007). Drought may lead to a decline in the water table, resulting in significant changes in the species competition level (Maestre et al., 2005), plant diversity, richness, evenness, coverage (Zeng et al., 2021), net primary productivity (Flanagan et al., 2013), litter yield, underground carbon distribution, and litter or secretion composition (Garssen et al., 2014). Changes in plant species composition and community structure may further affect the organic C pool of peatlands (De Deyn et al., 2008; Potvin et al., 2015). Re is divided into heterotrophic respiration generated by microbial decomposition residues and soil organic matter and plant autotrophic respiration or aboveground autotrophic respiration generated by a plant canopy and Rs. Different components may have different sensitivities to the same environmental factors (such as temperature and soil moisture), (Hu et al., 2015). Therefore, plant community changes after drought events may have a great impact on ecosystem C emissions, especially on Re and autotrophic respiration, but there is still a lack of relevant evidence on the specific influencing pathways.

It is well-known that soil extracellular enzyme activities (EEAs) provide a direct driving force for soil biogeochemical cycles, including C, nitrogen (N), and phosphorus (P) turnover (Burns et al., 2013). Full degradation in soil macromolecular organic matter depends on the synergistic effect among various soil extracellular enzymes, such as cellulose hydrolase and phenol oxidase, which can accelerate the decomposition of soil C (Freeman et al., 2004; Baldrian, 2009). Climate changes markedly alter soil EEAs *via* direct and indirect methods and in turn, modulate soil nutrient cycles (Henry, 2012; Wang et al., 2017a; Su et al., 2020). The activities of C-, N-, and P-acquisition enzymes have been proven to decrease significantly in the middle and late stages of the growing season under extreme drought (Gao et al., 2021). Water, oxygen, temperature, and pH interact to affect the activity of phenol oxidase, and the mineralization and output of peatland C are complex and sensitive to changes in environmental conditions (Laiho, 2006). Fenner et al. (2005), using microcosms, reported that simulated drought increased respiration, phenol oxidase, and β-glucosidase activities and decreased the concentration of phenolic. Few studies have explored the linkage between soil EEAs and ecological processes (Dominguez et al., 2017), which is a central focus in climate change ecology. Cellulose and lignin are the two most effluent soil organic matter (SOM) compounds. Meanwhile, microbial-mediated decomposition in the aforementioned substances is an important source of Re and Rs (Carreiro et al., 2000; Chen et al., 2018). The key role played by cellulase and lignase in mediating SOM decomposition reveals that extreme drought possibly affects Re and Rs *via* impacts on EEAs, but direct evidence of their contributions is still lacking.

Zoige peatlands cover an area of approximately 5,091 km^2^ of the Qinghai-Tibetan Plateau, and the corresponding peat C stock was reported to be 0.477 Pg (0.206–0.672 Pg), accounting for 88% of the total C storage of the Qinghai-Tibetan Plateau (0.543 Pg C), (Chen et al., 2014). The Zoige peatlands are major C sources of CO_2_ emissions due to the large C pool, potentially causing positive feedback to extreme drought. Thus, the Zoige peatlands provide a proper site for investigating extreme drought-induced variations in C emissions and the correlation of plants and EEAs with C emissions during extreme drought. The research tested Re and Rs responses after 5 years of simulated extreme drought events and relevant potential mechanisms. The study mainly answers three questions as follows: (i) What about experimental extreme drought impacts on Re and Rs dynamics? (ii) Do soil EEAs change significantly after extreme drought events? (iii) How do plants and soil EEAs regulate Re and Rs changes under extreme drought?

## Materials and Methods

### Study Area Experimental Design

The peatland investigated in this research is at an altitude of 3,430 m. The study area is situated on the Zoige Plateau (33°47′56.61″N, 102°57′28.43″E) of the northeastern Qinghai-Tibet Plateau. Due to the unique climatic and hydrological conditions of the region, the largest alpine peatlands worldwide have been discovered across the region (Ma et al., 2016). The average annual temperature ranges from −1 to 3.3°C, and the average annual precipitation is between 650 and 750 mm. The region has a long frost period (October to April) and a relatively short growing season (May to September), (Kang et al., 2016). The relevant average peat profile thickness across the region is approximately 1.39 m, and the maximum thickness can reach 10 m (Chen et al., 2014). The soil organic carbon (SOC) content fell between 179.36 and 276.41 g·kg^−1^. Soil pH was between 6.8 and 7.2. The dominant plant species are *Koeleria tibetica, Carex meyeriana, Carex muliensis, Blysmus sinocompressus, Eriophorum gracile*, and *Carex secbrirostris*.

We have collected precipitation statistics for the region for the past 5 decades (China Meteorological Data Service Center) and focused on one extreme drought event, not long-term drought, so fitting a Gumbel I distribution to the ~50-year local weather data. Daily precipitation ≤ 3 mm indicates an absence of precipitation. According to the definition of extreme climate (Hoover and Rogers, 2016), meteorological drought is defined as the number of consecutive days of precipitation deficiency compared with a long-term average (Mishra and Singh, 2010; Sheffield and Wood, 2012), and invalid precipitation lasting for 32 days is considered an extreme drought event for this region. The artificial extreme drought event simulation started in June 2014, and precipitation was prevented by transparent awning, which was 2.5 m in length, 2.5 m in width, and 1.8 m in height. The light transmittance of the shielding material is over 90%. Each treatment consisted of three plots (each plot was 2 m × 2 m), a total of 12 plots were established, and replicates had random distribution throughout the field. A 1-m-deep aluminum plate was inserted at the edge of the plot to prevent the horizontal flow of water. For more detailed information, refer to Kang et al. (2018) and Yan et al. (2020). After 5 years of continuous extreme drought event stress once a year, the experiment in 2019 included the control treatment (CK), which was exposed to ambient precipitation throughout the entire experiment and different periods of extreme drought events for approximately 32 days during the growing season. The three extreme drought treatments of different periods were imposed on June 18 to July 20 (ED: early drought), July 20 to August 23 (MD: midterm drought), and August 23 to September 25 (LD: late drought).

### Measurements of CO_2_ Fluxes

Ecosystem respiration and soil respiration were measured approximately one time a week between 10:00 and 15:00 (local time) from June 18 to September 25, 2019. The square aluminum frame (0.5 m × 0.5 m) was placed in the soil on every plot at 2-or 3-cm depth, providing a certain flat base between the soil surface and the CO_2_ sampling chamber. The experiment assessed Re using a 0.125-m^3^ opaque chamber (0.5 m × 0.5 m × 0.5 m) fixed onto a microportable greenhouse gas analyzer (GLA131, ABB, Canada), covering all vegetation within the aluminum frame. During the measurements, the air inside the chamber was mixed by two small fans. A previous study showed that this method can be successfully applied to the measurement of CO_2_ flux in alpine meadows (Chen et al., 2016). The CO_2_ concentration was recorded continuously for 120 s after the steady state was reached (steady-state conditions were typically achieved in the range of 10 to 30 s). Increases in air temperature were <0.2°C inside the chamber within measurement intervals (Xia et al., 2009). Polyvinylchloride (PVC) collars 20 cm in diameter and 10-cm high were placed in soil at 2-or 3-cm depth near the aluminum frames, which were used in Rs measurements. To eliminate the effects of plant respiration, live plants within soil collars were interrupted on the soil surface. A soil CO_2_ flux chamber (SC-12, LICA, China) attached to an infrared gas analyzer (PS-9000, LICA, China) was placed on each collar and then moved to the next collar after the automatic Rs measurement was completed.

Re (mg·m^−2^·h^−1^) can be calculated by a linear slope, which represents variations in gas concentration over time, which can be given by (Mastepanov et al., 2008).


(1) $$\begin{array}{r} Re=\frac{dc}{dt}\frac{M}{V_{0}}\frac{P}{P_{0}}\frac{T_{0}}{T}H \end{array}$$

*dc/dt* indicates the gas concentration slope with time (ppm·h^−1^); M suggests the molar mass for gas to be calculated (g·mol^−1^); *P* implies the atmospheric pressure of the sampling site (Pa); *P*_0_, *V*_0_, and *T*_0_ represent the standard atmospheric pressure (101,325 Pa), standard molar volume (22.41 m^3^·mol^−1^), and absolute temperature in standard atmospheric pressure; and *T* and *H* represent the absolute temperature (°C) and effective height (m) inside the chamber, respectively.

Rs (mg·m^−2^·h) is also calculated by the linear slope, which represents the change in gas concentration over time, which can be given by


(2) $$\begin{array}{r} Rs=\frac{10VP_{0}\left(1-\frac{W_{0}}{1000}\right)}{RS\left(T_{0}+273.15\right)}\frac{\partial C^{\prime }}{\partial t}43.2 \end{array}$$

*V* indicates the volume inside the air chamber; *P*_0_ means the standard atmospheric pressure (101,325 Pa), *W*_0_ is the initial water vapor value; *R* is a constant (8.314); *S* is the gas measurement area; *T*_0_ is the measured temperature during sampling; and ∂*C*′/∂*t* suggests the gas concentration slope over time (ppm·h^−1^).

### Plant and Soil Characteristics

Plant and soil samples (0–10 cm) of every plot were gathered on July 20, August 23, and September 25, 2019, at the end of each extreme drought event. Standing biomass (SBM) samples were gathered using a square frame (0.5 m × 0.5 m) near the aluminum frames, followed by drying at 65°C for 72 h prior to weighing. At the same time, the vegetation coverage and species number in different treatment plots were recorded. Soil samples collected from the five cores were stored in separate insulated bags and instantly delivered to the laboratory using ice boxes. Overall, fresh field soils fell into three subsamples, following screening *via* a 2-mm sieve for soil physical and chemical properties and soil EEA analysis. Altogether, 18 soil samples were investigated for each indicator. The soil water content (SWC) could be assessed *via* aTDR 300 moisture meter (Spectrum, USA) on site. Soil pH could be assessed through a pH electrode (PB-10, Sartorius, Germany) within a 1:2.5 soil-to-deionized water mixture. SOC, as well as total N (TN) concentrations, could be assessed through the C/N analyzer (Elementar, Vario Max CN, Germany) through dry combustion of samples (100-mesh). Microbial biomass C (MBC), microbial biomass N (MBN), and microbial biomass phosphorus (MBP), (Chen et al., 2016) were determined by an elemental TOC analyzer (LiquiTOC II, Germany), using the chloroform fumigation method. Dissolved organic carbon (DOC) was calculated for soil water extracts. A fresh sample of 15 g was placed in 150 ml of ultrapure water, oscillated at 15°C for 24 h, screened using a prebaked 0.7-mm glass fiber filter (GF/F, Whatman, UK), and then analyzed by an elemental TOC analyzer (LiquiTOC II, Germany). Soil NH${}_{4}^{+}$ and NO${}_{3}^{-}$ concentrations were determined by an auto analyzer (SEAL-AA3, Germany) from 2-mol L^−1^ KCl extracts. Total phosphorus (TP) was measured using a flow injection auto analyzer, followed by digestion through H_2_SO_4_-HClO_4_ (Huang et al., 2011). Available phosphorus (AP) was measured using molybdenum blue colorimetry with a spectrophotometer (TAS-990, Persee, Beijing).

### Monitoring Changes in Soil Enzyme Activities Under Extreme Drought

In this study, a total of seven soil hydrolases were determined to be engaged with C-, N-, and P-acquisition (Table 1). Soil hydrolases in α-1,4-glucosidase (AG), β-1,4-glucosidase (BG), β-1,4-xylosidase (BX), β-D-cellobiosidase (CB), β-1,4-N-acetyl-glucosaminidase (NAG), leucine amino peptidase (LAP), and alkaline phosphatase (ALP) were determined using a fluorescence method (Jackson et al., 2013). The first step was to add 50-μl, 200-mM substrate solution into every sample well, a 50-ml acetate buffer, 200-ml sample suspension to blank wells, 50-ml substrate solution, and a 200-ml acetate buffer into negative control wells. Then, quench standard wells accepted 50 ml of the standard, which is 10-mM 7-amino-4-methyl coumarin in LAP and 4-methylumbelliferone in other soil hydrolases, with 200 ml of the sample suspension. The reference standard wells accepted 50 ml of the standard and 200 ml of an acetate buffer. For every blank, negative control, and quench standard, there were eight replicate wells. Then, microplates needed to be cultivated in darkness at 20°C for 3 h. When the incubation period terminated, each well received a 10-ml aliquot of 1-M NaOH for stopping the reaction. Finally, fluorescence was assessed with a microplate fluorometer, containing 365-nm excitation and 450-nm emission filters.

**Table 1:** Description of soil extracellular enzymes in this study.

**Extracellular enzyme**	**EC**	**Type**	**Targets**
α-1,4-glucosidase	3.2.1.20	C-acquisition hydrolysis	Starch and disaccharides
β-1,4-glucosidase	3.2.1.21	C-acquisition hydrolysis	Cellulose
β-1,4-xylosidase	3.2.1.37	C-acquisition hydrolysis	Hemicellulose
β-D-cellobiosidase	3.2.1.91	C-acquisition hydrolysis	Cellulose
β-1,4-N-acetyl-glucosaminidase	3.1.6.1	N-acquisition hydrolysis	Chitin
Leucine amino peptidase	3.4.11.1	N-acquisition hydrolysis	Protein
Alkaline phosphatase	3.1.3.1	P-acquisition hydrolysis	Organic phosphorus
Polyphenol oxidase	1.10.3.1	Recalcitrant C oxidation	Lignin
Peroxidase	1.11.1.7	Recalcitrant C oxidation	Lignin

Soil oxidases of polyphenol oxidase (PPO) and peroxidase (PEO) were assessed spectrophotometrically with the substrate L-3,4-dihydroxyphenylalanine (DOPA), (Pind et al., 1994; Sinsabaugh, 2010). Every sample accepted 50 ml of 25-mM DOPA and 50 ml of 25-mM DOPA with 10 ml of 0.3% H_2_O_2_ in PPO and PEO measurements. Negative control wells received 200 ml of an acetate buffer as well as 50 ml of DOPA solution for PPO, and blank wells included 200 ml of sample suspension and 50 ml of the acetate buffer. For PEO, an extra 10 ml of H_2_O_2_ needed to be inserted into the negative control and blank wells. Altogether, 16 replicate sample wells were applied in every measurement, and eight replicate wells were adopted for the blank and control. Microplates were cultivated in darkness at 20°C for 18 h. The activity could be quantified by assessing the absorbance at 450 nm with a microplate spectrophotometer. The activity in soil hydrolases and oxidases was indicated by units of nmol g^−1^ soil h^−1^ upon correction in the negative control as well as quenching.

### Statistical Analyses

Repeated measures analysis of variance was adopted to compare the impacts exerted by extreme drought, sampling time, and their interactions on Re and Rs. Independent sample *t*-tests were conducted to determine how extreme drought treatment affected vegetation, soil characteristics, and soil EEAs. Significant differences were evaluated at the *p* < 0.05 level. The structural equation model (SEM) could be used separately to determine the impacts of different variables on Re and Rs under extreme drought. The maximum likelihood estimation approach was employed for fitting data. The model showed fine fitting effects if 0.05 < *p* ≤ 1.00 in a chi-square (χ^2^) test, comparative fit index (CFI) ≥ 0.95, and root mean square error of approximation (RMSEA) ≤ 0.05. Statistical analysis was performed with SPSS 22.0 (SPSS, Chicago, IL, USA); SEM analysis was performed with IBM SPSS Amos 24.0 (SPSS, Chicago, IL, USA). The figures were generated with OriginPro (Origin lab Corporation) and Adobe Illustrator CC (Adobe Inc.).

## Results

### Meteorological Conditions, Vegetation, and Soil Variables

**Figure 1:**
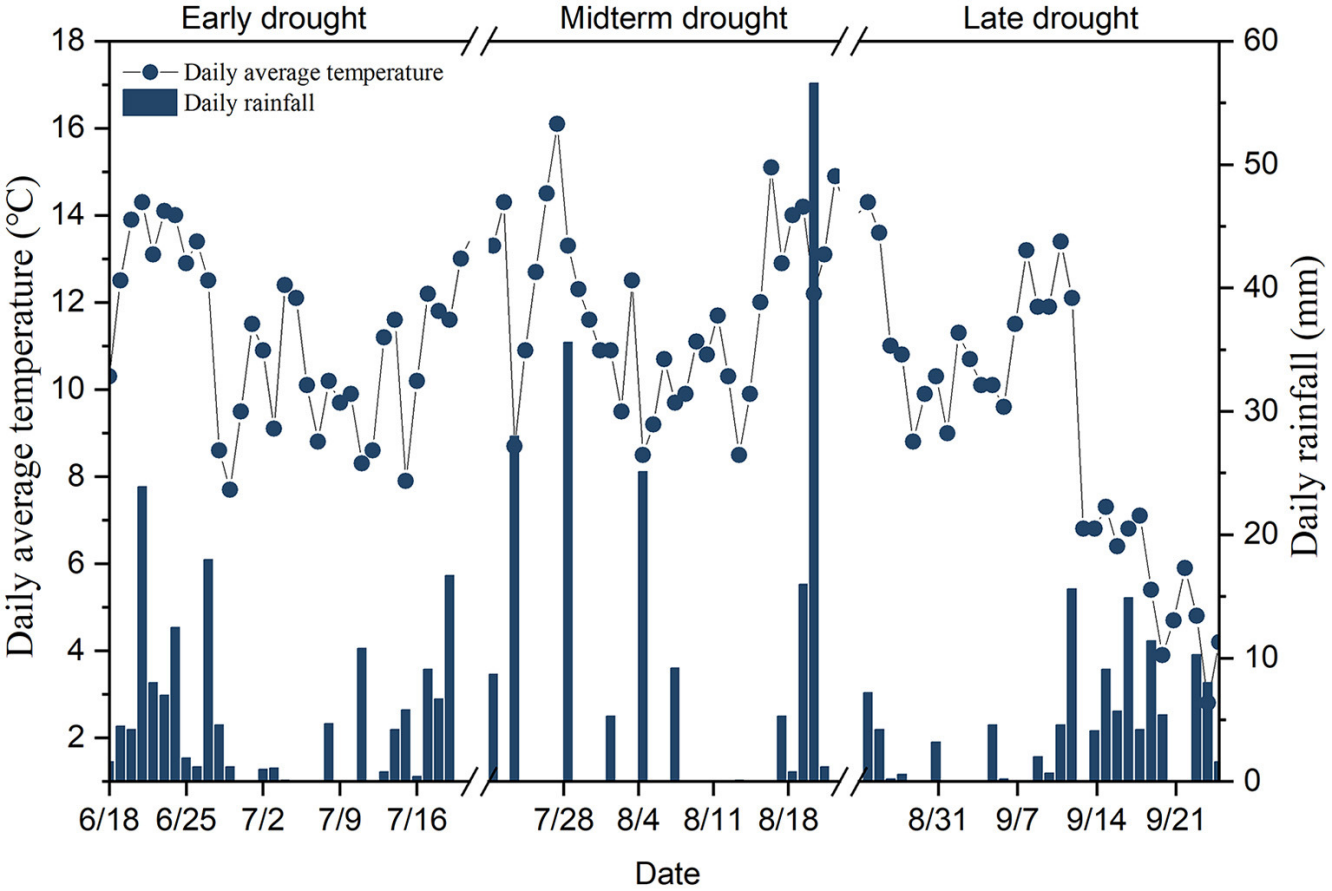
Average daily temperature and precipitation of experiments in 2019.

There was seasonal variation in the mean daily air temperature during the three extreme drought events, ranging from 2.8 to 16.1°C (Figure 1). The mean daily air temperature was highest on July 28 during the MD treatment and lowest on September 24 during the LD treatment. The mean daily air temperatures for the ED, MD, and LD treatments were 11.15, 11.89, and 9.10°C, respectively. There was also seasonal variation in daily precipitation during the three extreme drought events, ranging from 0 to 56.6°C. Daily precipitation was highest on August 21 during the MD treatment. The total precipitation for the ED, MD, and LD treatments was 150, 196.5, and 117.8 mm, respectively. The precipitation during the LD treatment was < that during the ED and MD.

The SBM decreased by 0.68, 18.33, and 2.38% compared with the control under the ED, MD, and LD treatments, respectively, and reached a significant level (*p* < 0.05) after MD (Table 2). Plant coverage increased by 79.31, 17.24, and 34.48% compared with the control under the ED, MD, and LD treatments, respectively, and reached a significant level (*p* < 0.05) under the ED treatment. The number of plant species showed no significant differences in treatments in 2019, decreased by 5.56% in the ED treatment, and increased by 5.56% under both MD and LD treatments in contrast to the control.

**Table 2:** Plant and soil characteristics in the control period and different periods of extreme drought in 2019.

**Variables**	**Early drought**		**Midterm drought**		**Late drought**	
	**CK**	**ED**	**CK**	**MD**	**CK**	**LD**
SBM (g m^−2^)	351.77 ± 16.00a	349.4 ± 9.99a	351.44 ± 15.68a	287.04 ± 9.26b	350.77 ± 16.73a	342.41 ± 24.58a
Coverage (%)	48.33 ± 8.33a	86.67 ± 3.33b	48.33 ± 8.33a	56.67 ± 8.33a	48.33 ± 8.33a	65 ± 8.66a
Species no.	6 ± 0a	5.67 ± 0.67a	6 ± 0a	6.33 ± 0.88a	6 ± 0a	6.33 ± 0.58a
SWC (%)	60.13 ± 0.33a	54.47 ± 1.88b	61.7 ± 0.8a	59.37 ± 1.55a	69.63 ± 0.5a	66.7 ± 1.45a
pH	7.73 ± 0.07a	7.83 ± 0.03a	7.73 ± 0.03a	7.87 ± 0.09a	7.8 ± 0.06a	7.9 ± 0.06a
SOC (g kg^−1^)	215.42 ± 19.75a	203.59 ± 28.56a	241.2 ± 6.59a	218.48 ± 21.01a	257 ± 13.18a	248.16 ± 13.14a
DOC (mg kg^−1^)	293.93 ± 14.86a	300.55 ± 12.98a	305.49 ± 78.29a	359.57 ± 72.99a	374.89 ± 14.72a	379 ± 22.8a
MBC (mg kg^−1^)	394.69 ± 59.35a	385.56 ± 53.63a	441.9 ± 4.54a	383.66 ± 55.62a	444.78 ± 11.14a	446.11 ± 7.09a
TN (g kg^−1^)	15.51 ± 0.48a	16.33 ± 0.15a	17.43 ± 6.37a	22.63 ± 3.45a	25.7 ± 1.85a	26.87 ± 2.87a
NH${}_{4}^{+}$ (mg kg^−1^)	11.69 ± 0.17a	11.05 ± 0.79a	11.91 ± 0.11a	11.95 ± 0.36a	12.4 ± 0.33a	12.45 ± 0.22a
NO${}_{3}^{-}$ (mg kg^−1^)	6.36 ± 0.77a	4.75 ± 1.38a	6.55 ± 0.31a	6.17 ± 0.15a	6.55 ± 0.35a	6.88 ± 1.22a
MBN (mg kg^−1^)	73.41 ± 1.49a	71.54 ± 1.36a	70.39 ± 1.94a	74.03 ± 1.7a	73.4 ± 1.77a	75.08 ± 0.22a
TP (g kg^−1^)	1.3 ± 0.06a	1.49 ± 0.11a	1.43 ± 0.03a	1.5 ± 0.05a	1.46 ± 0.08a	1.19 ± 0.29a
AP (mg kg^−1^)	40.5 ± 2.32a	33.46 ± 9.64a	23.9 ± 5.1a	29 ± 5.67a	43.03 ± 4.6a	57.06 ± 3.98a
MBP (mg kg^−1^)	34.15 ± 6.97a	31.9 ± 6.65a	39 ± 0.79a	35.57 ± 3.05a	39.5 ± 1.93a	39.73 ± 1.23a

*SBM, standing biomass; Species No., number of species; SWC, average soil water content during each extreme-drought period; SOC, soil organic carbon; DOC, dissolved organic carbon; MBC, microbial biomass carbon; TN, soil total nitrogen; MBN, microbial biomass nitrogen; TP, soil total phosphorus; AP, available phosphorus; MBP, microbial biomass phosphorus; ED, early drought; MD, midterm drought; LD, late drought; CK, the ambient control treatment corresponding to each stage of extreme-drought treatment. Different letters indicate a remarkable difference between the control and extreme drought treatments (p <0.05). Relevant data are indicated by the mean ± standard error (n = 3)*.

The soil water content decreased by 9.42, 3.87, and 4.21% compared with the control under the ED, MD, and LD treatments, respectively, and reached a significant level (*p* < 0.05) during the ED treatment (Table 2). Soil pH, DOC, and TN increased by 1.29, 2.25, and 5.29% under the ED treatment, by 1.73, 17.70, and 29.83% under the MD treatment, and by 1.28, 1.10, and 4.54% under the LD treatment, respectively. SOC decreased by 5.49, 9.42, and 3.44% under the ED, MD, and LD treatments, respectively. MBC and MBP decreased by 2.31 and 6.61% under ED treatment, decreased by 13.18 and 8.79% under MD treatment, and increased by 0.30 and 0.58% under LD treatment, respectively. NH${}_{4}^{+}$, MBN, and AP decreased by 5.53, 2.55, and 17.37% under the ED treatment, increased by 0.31, 5.18, and 21.34% under the MD treatment, and increased by 0.43, 2.29, and 32.61% under the LD treatment, respectively. NO${}_{3}^{-}$ decreased by 25.35 and 5.90% under the ED and MD treatments, respectively, but increased by 5.04% under the LD treatment. TP increased by 14.36 and 4.65% under ED and MD treatments, respectively, and decreased by 18.72% under LD treatment.

### Effects of Extreme Drought on Re and Rs Dynamics

**Figure 2:**
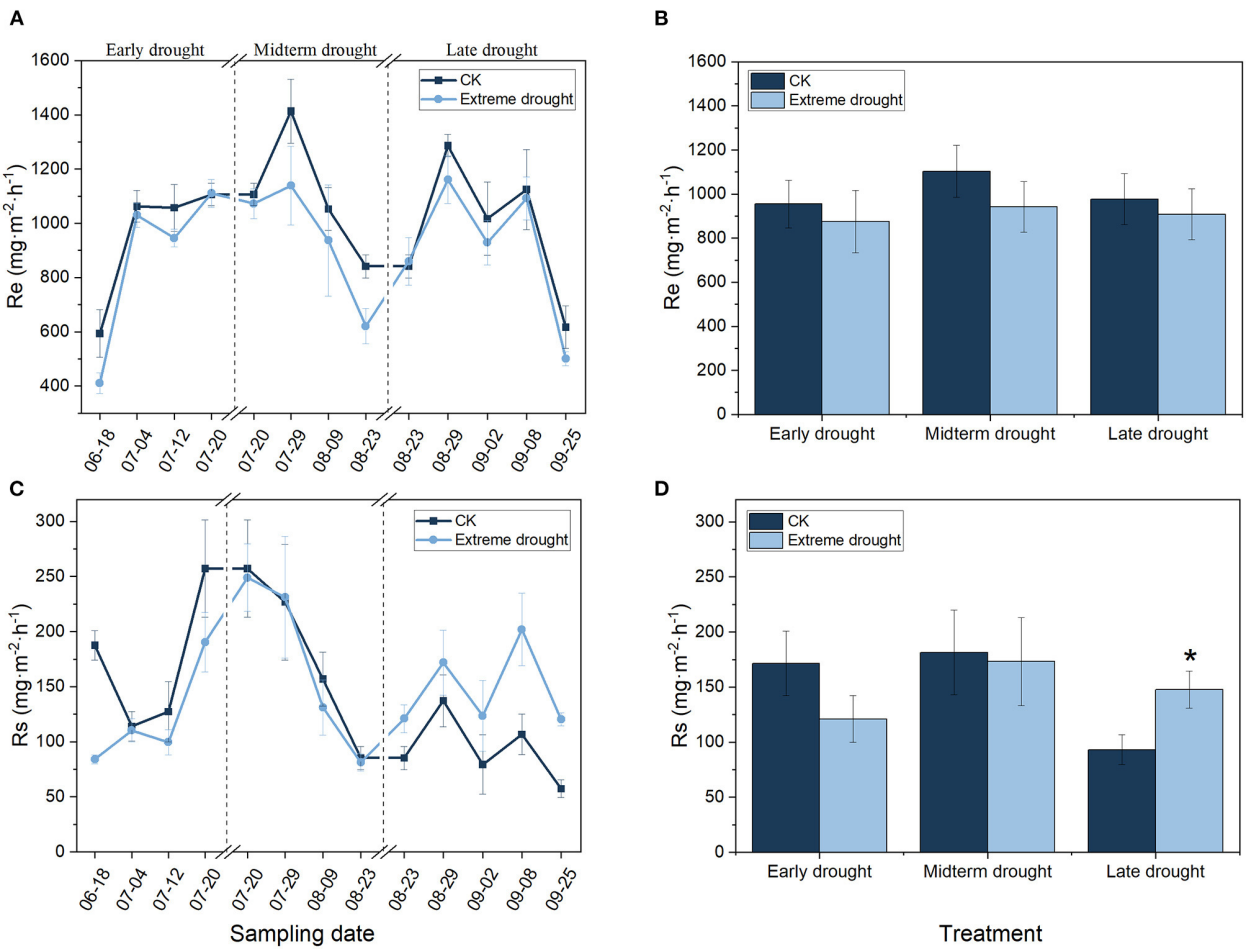
Seasonal variations in Re **(A,B)** and Rs **(C,D)** in the growing season of 2019 for different periods of extreme drought treatments (*n* = 3; bars indicate SE). Significance: *p < 0.05.

Ecosystem respiration and soil respiration showed similar seasonal patterns in the control and three different periods of the extreme drought treatments in 2019 (Figures 2A,C). The Re ranged from 410.51 to 1286.49 mg·m^−2^·h^−1^ in the growing season and reached its highest values at the end of July; additionally, when the growing season began and terminated, Re was lower than that at the other sampling times. The seasonal mean Re decreased by 8.46 (874.10 mg·m^−2^·h^−1^), 14.59 (942.41 mg·m^−2^·h^−1^), and 7.03% (908.48 mg·m^−2^·h^−1^) during the ED, MD, and LD treatments, respectively, in contrast to the control (Figures 2A,B). Rs ranged from 57.34 to 257.17 mg·m^−2^·h^−1^ during the growing season, reaching its highest value until mid-July. The seasonal mean Rs decreased by 29.38 (121.09 mg·m^−2^·h^−1^) and 4.62% (173.12 mg·m^−2^·h^−1^) under the ED and MD treatments, respectively, but increased significantly (*p* < 0.05) by 58.48% (147.66 mg·m^−2^·h^−1^) under the LD treatment (Figures 2C,D).

Generally, Re and Rs were significantly influenced by the sampling date during 2019 (Table 3) in the different periods of the three extreme drought treatments, and no remarkable interactions were found between the sampling date and drought treatment. Notably, Rs increased remarkably under the LD treatment (*p* < 0.05).

**Table 3:** The effects of different periods of extreme drought treatment, sampling time, and relevant interactions on ecosystem respiration (Re) and soil respiration (Rs) in 2019.

**Variance source**	**Early drought**			**Midterm drought**			**Late drought**		
	**df**	**Re**	**Rs**	**df**	**Re**	**Rs**	**df**	**Re**	**Rs**
Treatment (T)	1	2.674	6.413	1	2.741	0.113	1	0.733	7.79^*^
Date (D)	4	53.092^***^	13.615^***^	4	11.006^**^	8.96^**^	4	22.58^***^	5.20^**^
T × D	4	1.194	2.358	4	0.62	0.15	4	0.321	0.841

*Significance*:

*** *, p <0.001*;

** *, p <0.01*;

* *, p <0.05. Re, ecosystem respiration; Rs, soil respiration*.

### Effects of Extreme Drought on Soil EEAs

**Figure 3:**
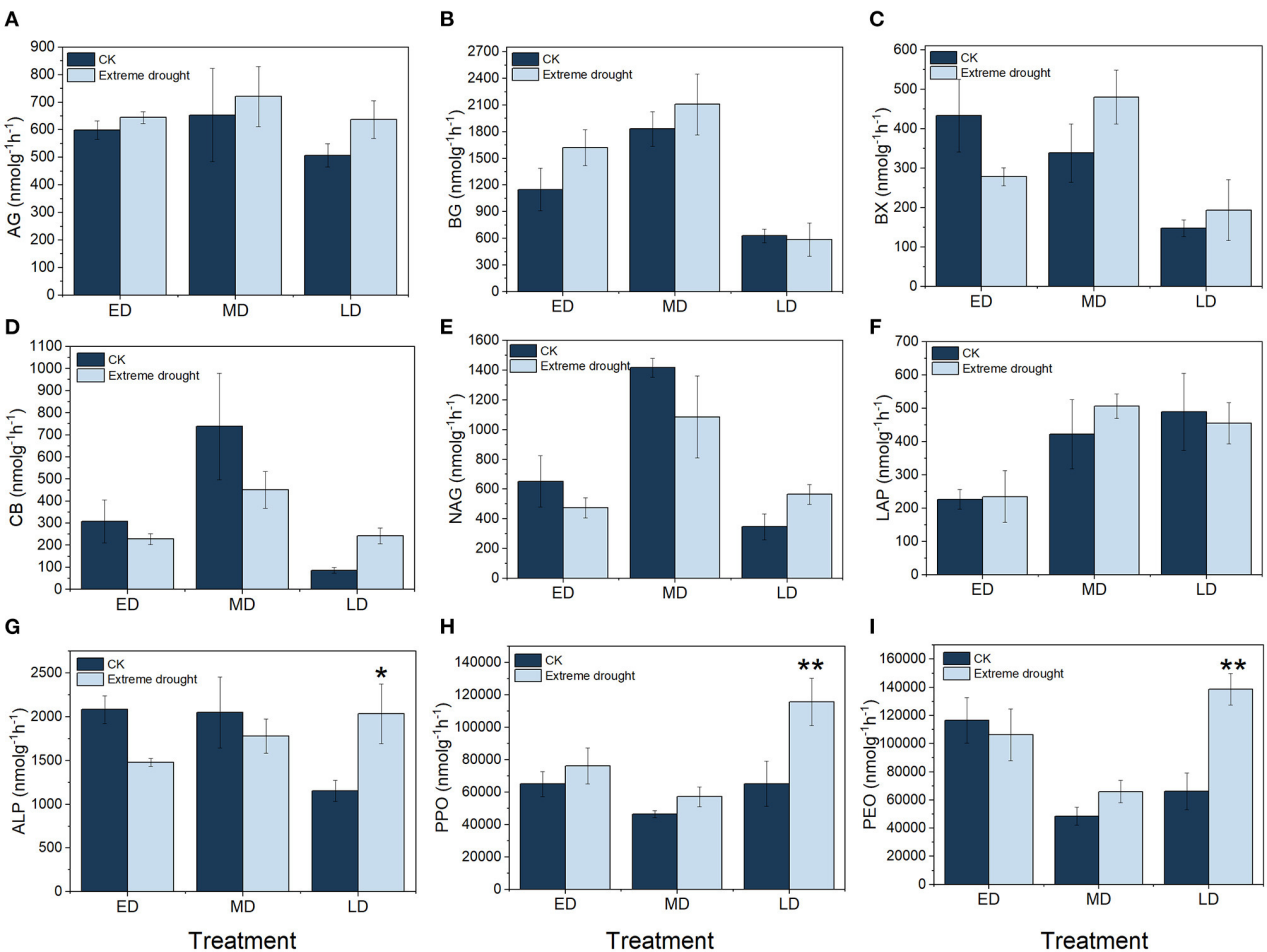
Soil enzyme activities of C-acquisition **(A–D,H,I)**, N-acquisition **(E,F)** and P-acquisition **(G)** resulting from different treatments. ED, early drought; MD, midterm drought; LD, late drought; CK, the ambient control treatment corresponding to each stage of extreme drought treatment. AG, α-1,4-glucosidase; BG, β-1,4-glucosidase; BX, β-1,4-xylosidase; CB, cellobiosidase; NAG, β-1,4-N-acetyl-glucosaminidase; LAP, leucine amino peptidase; ALP, alkaline phosphatase; PPO, polyphenol oxidase; PEO, peroxidase; **, *p* < 0.01; *, *p* < 0.05. Relevant data are indicated by the mean ± standard error (*n* = 3; bars indicate SE).

In various extreme drought periods, the soil EEAs showed different trends (Figure 3): BG, ALP, and PPO had the highest activities among all soil enzymes (Figures 3B,F,H); their ranges of variation were 587.27–2108.84 nmol g^−1^ h^−1^, 1152.80–2082.92 nmol g^−1^ h^−1^ and 46443.52–11560.30 nmol g^−1^ h^−1^, respectively. AG and PPO increased by 7.61 (644.54 nmol g^−1^ h^−1^) and 17.08% (76136.08 nmol g^−1^ h^−1^) in the ED treatment, 10.31 (721.09 nmol g^−1^ h^−1^) and 22.95% (57101.98 nmol g^−1^ h^−1^) in the MD treatment, and 25.49 (636.92 nmol g^−1^ h^−1^) and 77.66% (115660.30 nmol g^−1^ h^−1^) in the LD treatment, respectively, compared with CK (Figures 3A,H). BG and LAP increased by 40.95 (1620.24 nmol g^−1^ h^−1^) and 3.59% (234.89 nmol g^−1^ h^−1^) in the ED treatment, increased by 15.15 (2108.84 nmol g^−1^ h^−1^) and 19.80% (505.80 nmol g^−1^ h^−1^) in the MD treatment, and decreased by 6.51 (587.27 nmol g^−1^ h^−1^) and 7.08% (454.78 nmol g^−1^ h^−1^) in the LD treatment, respectively, compared with CK (Figures 3B,F). CB, NAG, and ALP decreased by 26.01 (228.08 nmol g^−1^ h^−1^), 27.19 (474.34 nmol g^−1^ h^−1^), and 29.03% (1478.25 nmol g^−1^ h^−1^) in the ED treatment, decreased by 38.86 (451.10 nmol g^−1^ h^−1^), 23.36 (1085.05 nmol g^−1^ h^−1^), and 13.23% (1779.06 nmol g^−1^ h^−1^) in the MD treatment, and increased by 181.18 (243.18 nmol g^−1^ h^−1^), 63.78 (565.34 nmol g^−1^ h^−1^), and 76.46% (2034.22 nmol g^−1^ h^−1^) in the LD treatment, respectively, compared with CK (Figures 3D,E,G). BX and PEO decreased by 35.72 (278.78 nmol g^−1^ h^−1^) and 8.76% (106366.50 nmol g^−1^ h^−1^) in the ED treatment, decreased by 41.85 (480.47 nmol g^−1^ h^−1^) and 30.83% (66000.25 nmol g^−1^ h^−1^) in the MD treatment, and decreased by 41.85 (480.47 nmol g^−1^ h^−1^) and 109.60% (138704.20 nmol g^−1^ h^−1^) in the LD treatment, respectively, compared with CK (Figures 3C,I). In the LD treatment, ALP, PPO, and PEO seemed remarkable (*p* < 0.05) > their counterparts in the control.

### Impact of Pathways of Extreme Drought on Re and Rs

**Figure 4:**
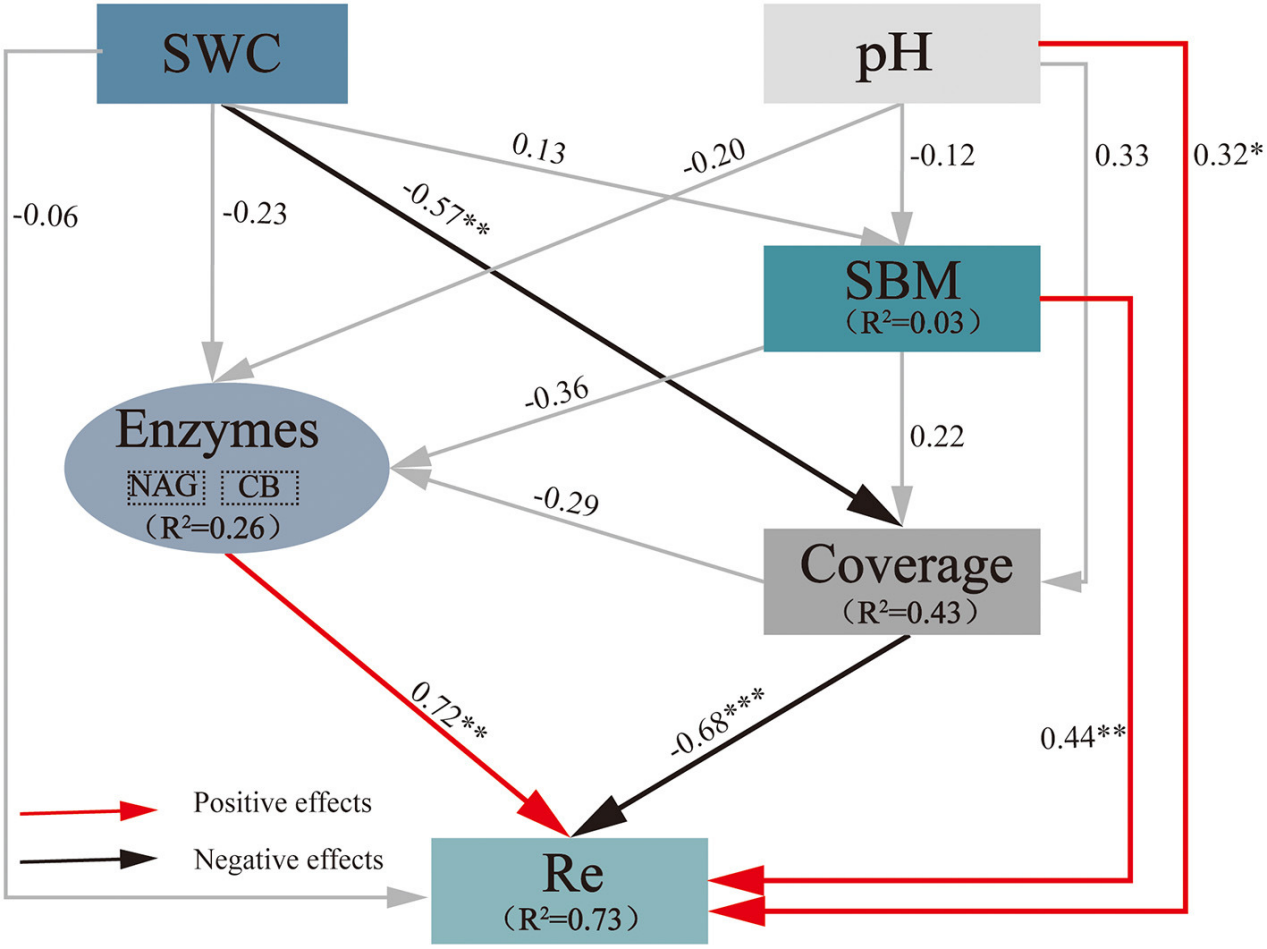
Impacts of selected drivers on Re under extreme drought calculated by the SEM of path analysis based on data collected in 2019. The values on the arrows are normalized path coefficients, and the asterisks after the numbers indicate remarkable correlations (*p < 0.05, **p < 0.01, and ***p < 0.001). The rectangle *R*^2^ value represents the ratio of variance explained by relationships with other variables. SWC, soil water content; SBM, standing biomass; NAG, β-1,4-N-acetyl-glucosaminidase; Re, ecosystem respiration.

An SEM was used to predict the effects of extreme drought on Re (Figure 4), with SWC, pH, NAG, CB, plant coverage, and standing biomass (SBM) as the variables (χ^2^ = 4.985, d*f* = 5, *P* = 0.418; CFI = 1.000; RMSEA = 0.000). The final model explained 73% of the variance in Re, 3% of that in SBM, 43% of that in coverage, and 26% of that in soil hydrolases (NAG and CB). SWC generated prominent direct negative effects on plant coverage (−0.57), and coverage generated prominent direct negative effects on Re (−0.68). pH (0.32), soil hydrolases (0.72), and SBM (0.44) generated an obvious direct positive effect on Re. These results implied that a decrease in SWC due to extreme drought resulted in a decrease in Re through plant coverage in the growing season, while the increase in pH, soil hydrolases, and SBM could result in an increase in Re. The final variation in Re is under the combined influence of the above factors.

**Figure 5:**
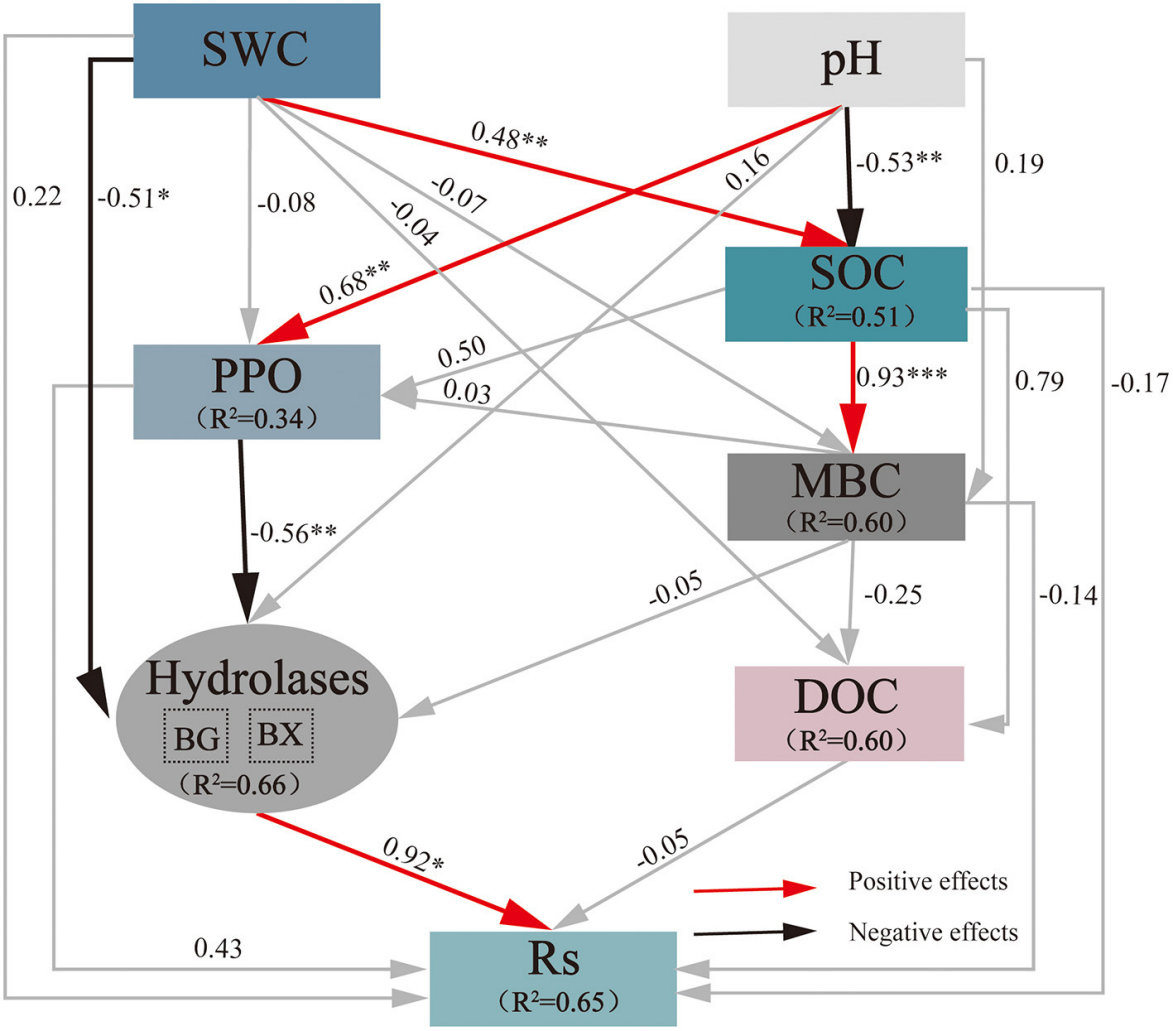
Impacts of selected drivers on Rs under extreme drought calculated by the SEM of path analysis based on data collected in 2019. The values on the arrows are normalized path coefficients, and the asterisks after the numbers indicate remarkable correlations (*p < 0.05, **p < 0.01, and ***p < 0.001). The rectangle *R*^2^ value represents the ratio of variance explained by relationships with other variables. SWC, soil water content; SOC, soil organic carbon; MBC, microbial biomass carbon; DOC, dissolved organic carbon; BG, β-1,4-glucosidase; Rs, soil respiration.

The final SEM for predicting the direct and indirect impacts exerted by drought on Rs included the following variables (Figure 5): SWC, pH, SOC, DOC, MBC, BX, BG, and PPO (χ^2^ = 7.992, d*f* = 11, *P* = 0.714, CFI = 1.000, RMSEA = 0.000). The final model explained 65% of the variance in Rs, 51% of that in SOC, 60% of that in DOC, 60% of that in MBC, 34% of that in PPO, and 66% of that in soil C-acquisition hydrolases (BG and BX). SEM demonstrated that SWC had a significant direct negative effect (−0.51) on soil C-acquisition hydrolases and a significant direct positive effect on SOC (0.48). Soil pH showed an obvious direct negative impact on SOC (−0.53) and a significant direct positive effect on PPO (0.68). PPO showed an obvious direct negative impact on soil C-acquisition hydrolases (−0.56), and soil C-acquisition hydrolases showed an obvious direct positive impact on Rs (0.92), but the other soil indexes showed no significant direct influences. These results indicated that a decrease in SWC due to extreme drought resulted in an increase in soil C-acquisition hydrolases, while an increase in pH also increased PPO activity and then resulted in a decrease in soil C-acquisition hydrolases. Their synthesis led to a variation in soil C-acquisition hydrolases and then regulated Rs changes.

## Discussion

### Different Responses of Re and Rs to Extreme Drought

Our results revealed that Re had a non-significant decreasing trend (Figures 2A,B) under early, midterm, and late extreme drought in the growing season compared with the ambient control, which may be explained by Bhanja and Wang (2021), which found SWC to be the least influential factor in respiration estimation. Re can be divided into several subcomponents, including heterotrophic respiration and above-and belowground plant autotrophic respiration (Sagar et al., 2019). These components respond to extreme drought in complex ways, and plant autotrophic respiration accounts for a high proportion of Re. Drought induces changes in plant biodiversity, richness, evenness, coverage, and standing biomass (Ma et al., 2020; Zeng et al., 2021). Therefore, vegetation conditions must be taken into account when estimating Re from peatlands, especially in future extreme drought conditions. In our study, after 5 years of extreme drought, despite some changes in plant biomass and coverage, the rewet after the end of extreme drought every year may be conducive to vegetation restoration, which likely caused the lower but not significant Re under early, midterm, and late drought compared with ambient conditions.

In the past 3 decades, as the second continental carbon flux, Rs has been the main way for carbon dioxide absorbed by terrestrial plants to return to the atmosphere, increasing the positive feedback of climate warming (Bond-Lamberty et al., 2018). As identified by several studies, Rs was mainly affected by soil temperature and moisture (Carey et al., 2016; Yu et al., 2021). Increased water stress caused by drought may lead to reduced C synthesis for photosynthesis, which reduces substrate availability and microbial activity, thus impeding microbial and plant root respiration, thereby affecting Rs (Davidson et al., 2012; Fuchslueger et al., 2014). However, other points of view have indicated that extreme drought may lead to restricted root growth as well as soil organic matter-related microbial decomposition, with significant decreases in Rs (Sheik et al., 2011). Liu et al. (2016) suggested that an increase in precipitation also leads to an increase in Rs across arid regions, and the relevant excitation rate possibly decreases as climate humidity increases. Our results revealed that Rs increased significantly under late drought (*p* < 0.05) but decreased not significantly under early and midterm drought (Figures 2C,D). The responses of Rs diverged in response to drought in different stages of the growing season because autotrophic and heterotrophic Rs respond asymmetrically to drought (Huang et al., 2018). More detailed process analysis is necessary to reveal the mechanisms.

Both Re and RS are generally classified into heterotrophic and autotrophic respiration source components (Zhou et al., 2019). Under the continuous input of plant-derived C, soil constitutes the largest terrestrial C pool, which plays a decisive role in the global C cycle (Witzgall et al., 2021). Therefore, these results have implications for ecosystem function and climate change adaptation. If precipitation pattern change in the region leads to extreme drought events in the late period of the growing season, rather than the early or midterm period, then a significant increase in Rs could disrupt the balance of soil-atmosphere CO_2_ exchange, resulting in a loss of soil C pools and triggering C-climate positive feedback. These results provide new insights into assessing the risks and benefits of extreme drought in alpine peatlands. In the future, a better understanding of the source components of Re and Rs will help to understand their overall response during different periods of extreme drought events.

### Effects of Extreme Drought on Soil Enzyme Activities

Previous studies have compared the effects of drought on soil EEAs, but the results are controversial. A meta-analysis indicated that a decrease in precipitation caused a prominent decrease in soil microbial biomass and oxidoreductase activity by 11.61 and 10.99%, respectively, while C-acquisition hydrolase activity increased by 25.79% (Ren et al., 2017). Geisseler et al. (2011) suggested that, under dry conditions, soil EEAs significantly increased. According to Freeman et al. (2004), drought in peatlands increases oxygen and oxidase activity, resulting in low abundance of phenolic materials, which can further stimulate BG and BX activities. Plant standing biomass and soil substrates are considered to affect the response of C-acquisition EEAs to extreme drought (Beier et al., 2012; Steinauer et al., 2015). Therefore, in different extreme drought periods, the difference in plant coverage may also cause different changes in soil C-, N-, and P-acquisition EEAs (Baldrian, 2009; Baldrian et al., 2013; Bowles et al., 2014). Our results showed that, in an alpine peatland, the effects of extreme drought on soil EEAs were not uniform when comparing the early, midterm, and late periods of the growing season. Extreme drought that occurred during the plant-wilting period remarkably promoted soil oxidoreductase activity by 77.66 (PPO) and 109.60% (PEO) and increased P-acquisition hydrolase (ALP) activity by 76.46%. In contrast, extreme drought did not affect C-acquisition hydrolase (AG, BG, BX, and CB) or N-acquisition hydrolase (NAG and LAP) activities. The significant increase in soil oxidoreductase activity typically involved in recalcitrant C turnover under late drought in this study is in line with many studies (Li et al., 2020; Su et al., 2020) that have found that drought accelerated the degradation and C loss of peatlands (Zeng et al., 2021).

Due to the difference in SWC and its impacts on soil microbial biomass and plant productivity, the response of soil EEAs to drought showed divergence at different periods of extreme drought. The reduction in SWC and SBM was not the highest in the late-drought treatment of 2019, while the MBC was increased compared with the control treatment, which signified changes in plant and microbial competition, thereby facilitating microbial physiological activity and promoting the secretion of soil extracellular enzymes by a range of bacteria and fungi (Falade et al., 2017). During the early and midterm drought periods, the soil DOC was lower than that during the late-drought period, which showed that there were seasonal differences in the C turnover rate in different periods. In addition, a decline in the soil extractable C supply was caused by extreme drought through their indirect impact on plant growth (Zhou et al., 2019), therefore resulting in different changes in soil EEAs. Massive SOC storage was locked up in peatlands, probably because of the high stabilized oxidative enzyme activity induced by higher soil moisture and anaerobic environments (Freeman et al., 2001), while the high-potential activity of oxidative enzymes accelerated the degradation of phenolic compounds and thereby, increased decomposition potentials (Sinsabaugh, 2010). In our study, a decline in SOC under early, midterm, and late drought was observed; in particular, the increased oxidase activities under the late-drought period may be linked to large amounts of soil C loss and threaten the C-sink function of peatlands in the future.

### Impact of Pathways of Extreme Drought on Alpine Peatland CO_2_ Efflux

Previous studies have suggested that drought may have a significant impact on the underground C dynamics of overwhelming terrestrial ecosystems (Suseela et al., 2014), which implied that extreme drought could alter the decomposition of SOM with corresponding consequences for Re. Here, with a field-manipulative extreme drought experiment using different influencing factors, an obvious positive correlation of soil hydrolases, including C-acquisition (CB) and N-acquisition (NAG), with Re (Figure 4) could be found, and this correlation should mainly come from the effect of soil hydrolases on the decomposition of SOM (Collins et al., 2008; Sinsabaugh, 2010). Because the autotrophic respiration of aboveground plants accounts for a large proportion of Re, we also observed that Re is significantly positively correlated with SBM and negatively correlated with plant coverage (Figure 4). Previous studies have shown that drought causes species to adapt to low-humidity habitats to replace species adapted to flooded habitats of different peatland types (Weltzin et al., 2000; Bakker et al., 2007). In the peatland of the Qinghai-Tibetan Plateau, the SBM decreased and remained unchanged in the 1st and 2nd years after the water table decreased and began to increase in the 3rd year. The increase in primary productivity is mainly due to the transformation of dominant aquatic plants into mesophytes (Cao et al., 2017; Wang et al., 2017b), and the duration of groundwater-level reduction has a great impact on the composition of the plant community. In this study, after the annual extreme drought event, there was still a rewet period to promote the restoration of vegetation, which was the same as the conclusion of short-term drought in the previous 2 years of the above studies. However, the change in vegetation community led to the increase in vegetation coverage, so SBM and plant coverage had different effects on ecosystem respiration, and plant coverage showed a significant and negative correlation with SWC (Figure 4). Although different periods of extreme drought did not cause significant changes in Re, under a series of complex influencing factors, Re showed a consistent downward trend in the early, midterm, and late extreme drought compared to the ambient control. It is worth noting that the investigation of the vegetation community should be strengthened, and different components of Re responses to extreme-drought events should be explored to reveal the multiple sources of Re variations.

The rate-limiting procedure of soil organic matter decomposition is catalyzed by soil extracellular enzymes; the activity of which plays a key role in determining Rs (Chen et al., 2018). Oxidative enzymes promote the oxidation and degradation of refractory compounds, containing phenols (Sinsabaugh, 2010), namely, PPO and PEO (Martinez et al., 2005). As a major component in the plant cell wall, the cellulose and hemicellulose hydrolase process has been primarily catalyzed by hydrolytic enzymes, such as BG, BX, and CB (Jian et al., 2016; Chen et al., 2017). This result conformed to former findings, the breakdown in organic matter and the release of CO_2_ from biogeochemical cascades by stimulating cellulose (BG) and hemicellulose (BX) hydrolase (Figure 5). Although oxidase activities are often considered to be uncorrelated with hydrolase activities (Sinsabaugh, 2010), the “enzymatic latch” hypothesis proposed by Freeman et al. (2001) showed that hydrolytic enzyme activities inhibited by phenolic compounds accumulate in peat because of the protection from degradation by oxidative enzymes. Our results do not support the concept of the “enzymatic latch” because PPO was significantly negatively correlated with soil hydrolase (BG and BX) (Figure 5). Recent studies have raised similar challenges for the “enzymatic latch” (Urbanova and Hajek, 2021). The reason may be that the peat microbial community seems to adapt to the natural high-phenol concentration, which is characteristic of other non-oceanic northern peatlands. In addition, we found that PPO activities showed a significant and positive correlation with soil pH, and similar results were also observed by Wu et al. (2021). Additionally, the negative relationships of soil hydrolase activities with SWC indicated that extreme drought may have the potential to stimulate soil hydrolases.

**Figure 6:**
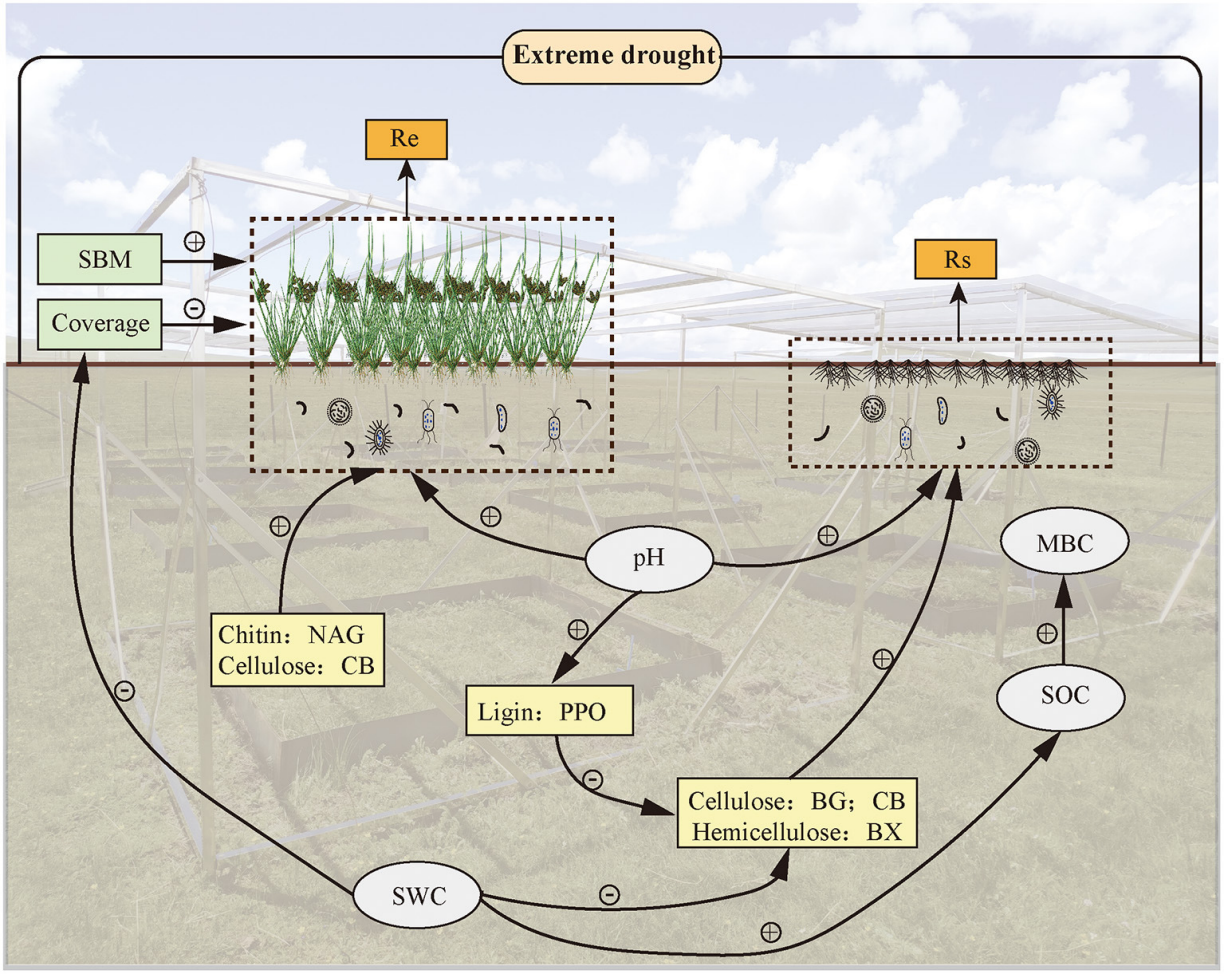
A conceptual framework outlining the direct effects of soil EEAs, soil physicochemical properties, and plant variables on ecosystem C dynamics under extreme-drought conditions.

To take a closer look at driving forces in regulating ecosystem CO_2_ release, we analyzed the influence of Re and Rs on changes in plants and soil EEAs during extreme drought (Figure 6). At present, the characterization of soil organic matter decomposition under soil EEAs regulation is lacking in the model prediction of ecosystem C dynamics (Davidson and Janssens, 2006; Wieder et al., 2013; Luo et al., 2016). However, we found that extreme drought-induced changes in soil hydrolases, as well as oxidases, possibly stimulate a continuous increase in Rs at the end of plant growth under repeated extreme drought conditions, which suggests the need to integrate enzymatic decomposition with soil biogeochemical models, and the mechanisms that regulate the effects in different periods remain to be elucidated. Furthermore, soil extracellular enzymes that are mainly produced by microorganisms play an important role in the decomposition of SOM, which also shows the importance of soil microorganisms in soil C turnover (Van Bodegom et al., 2005; Fenner and Freeman, 2011).

## Conclusion

In the current study, the responses of the factors we measured to extreme drought were inconsistent; Re, Rs, and soil EEAs exhibited no significant change in the early and midterm drought periods of the growing season compared with the ambient control, while plant coverage significantly increased in the early drought and SBM significantly decreased in the midterm drought. The activities of soil oxidases and P-acquisition enzymes, as well as Rs, were significantly enhanced under late drought. The results of the structural equation model suggested that SWC, pH, NAG, CB, plant coverage, and SBM were the main forcing variables affecting Re dynamics, and SWC, pH, SOC, DOC, MBC, BX, BG, and PPO were the main forcing variables affecting Rs dynamics. Overall, this study provides experimental evidence that extreme drought occurring during the plant-withering period increased Rs, which altered the CO_2_ balance between the soil and atmosphere in alpine peatlands. The regulation of plants and soil EEAs during Re and Rs changes suggested their important roles in the process of determining alpine ecosystem C emissions. The above findings help gain insights into C-climate feedback under future climate change and provide scientific evidence to maintain the functions and sustainability of the alpine peatland ecosystem. In future research, it is necessary to investigate and explain the impact mechanisms of the species composition of vegetation, as well as the resistance and resilience of alpine peatland ecosystem functions to extreme drought events.

## Data Availability Statement

The raw data supporting the conclusions of this article will be made available by the authors, without undue reservation.

## Author Contributions

ZY, YL, YH, and XK: contributed to the conception and the design of the study. EK and HW: organized the database. ZY and KZ: performed the statistical analysis. ZY and XK: wrote the first draft of the manuscript. ML, XZ, JW, and LY wrote sections of the manuscript. All the authors contributed to manuscript revision and read and approved the submitted version.

## Funding

This study was supported by the National Non-profit Institute Research Grant (CAFYBB2019SY030), the National Natural Science Foundation of China (32171597 and 42041005), and the National Non-profit Institute Research Grant (CAFYBB2020ZA004).

## Conflict of Interest

The authors declare that the research was conducted in the absence of any commercial or financial relationships that could be construed as a potential conflict of interest.

## Publisher's Note

All claims expressed in this article are solely those of the authors and do not necessarily represent those of their affiliated organizations, or those of the publisher, the editors and the reviewers. Any product that may be evaluated in this article, or claim that may be made by its manufacturer, is not guaranteed or endorsed by the publisher.
